# TerrainFormer: World Model-Guided Decision Transformer for Autonomous Off-Road Navigation

**DOI:** 10.3390/s26123795

**Published:** 2026-06-14

**Authors:** Yongzhi Yang, Kenneth Ricks

**Affiliations:** Department of Electrical and Computer Engineering, The University of Alabama, Tuscaloosa, AL 35487, USA; kricks@eng.ua.edu

**Keywords:** autonomous navigation, off-road robotics, world models, decision transformers, LiDAR perception, point cloud processing, behavioral cloning, terrain traversability, temporal reasoning, cross-dataset generalization

## Abstract

Autonomous navigation in unstructured off-road environments presents fundamental challenges due to terrain heterogeneity, the absence of structured road markings, and the necessity for real-time traversability reasoning from raw sensory observations. We present TerrainFormer, a hierarchical framework that integrates a world model for terrain dynamics prediction with a temporal decision transformer for action selection. Our methodology employs a two-phase training paradigm: (1) self-supervised world model pretraining on LiDAR point clouds to learn terrain representations encompassing traversability, elevation, and semantic segmentation; (2) behavioral cloning of the decision transformer conditioned on frozen world model features with temporally derived goal directions. The world model processes raw 3D LiDAR point clouds through a PointPillars encoder for real-time bird’s-eye-view (BEV) projection, followed by a Vision Transformer backbone that produces latent terrain representations. A principal contribution is our cross-dataset generalization paradigm: the world model is trained on separate datasets while the decision transformer is trained on separate sequences, ensuring zero data overlap between training phases. We introduce automatic goal direction computation from vehicle pose trajectories, enabling the model to learn directionally conditioned navigation policies. To address the class imbalance inherent in off-road driving data, we employ focal loss with inverse-frequency class weighting and action-chunk supervision. Experimental evaluation on the RELLIS-3D dataset achieves 87.31% test accuracy with 0.7948 macro F1 across all 12 action classes. The world model’s predicted future frames produce only a 0.79% accuracy drop versus ground-truth observations, with 98.82% action agreement, demonstrating effective cross-dataset generalization for real-time off-road navigation.

## 1. Introduction

Off-road navigation is the open problem behind a long list of robotic-vehicle applications: search and rescue, agriculture, military logistics, and planetary exploration. The hard part is the same in every case. Without lane markings or traffic infrastructure, the robot has to decide whether the ground in front of it is safe to drive on by looking at raw sensor data, in real time, while it moves [1,2]. The terrain surface itself varies from packed dirt to mud to loose gravel to dense vegetation, often within a single traversal.

The classical answer is a stack of hand-engineered modules: a terrain classifier, a planner, a controller [3]. Each module works on a constrained domain and fails outside it. Deep learning has shifted the perception layer of this stack from hand-engineered features to learned ones [4,5], but most of the deep learning literature still treats perception and planning as separate problems and trains them with separate objectives. The cost of that decoupling is that the perception model is not optimized for the eventual driving task.

Two recent threads make a different design possible. World models [6,7,8] compress environment dynamics into a low-dimensional latent that supports forward prediction, so the same backbone can learn terrain structure and predict its evolution. Decision transformers [9,10] cast offline policy learning as sequence modeling, so the same transformer machinery used for language can be aimed at action prediction [11]. Combining them puts terrain understanding and action selection inside one architecture without forcing them to share weights.

This paper describes TerrainFormer, the system that results from that combination. Specifically:

- A world model that takes raw 3D LiDAR point clouds and is pretrained self-supervised on four prediction targets at once: future BEV reconstruction, traversability, elevation, and semantic segmentation.
- A decision transformer with action chunking [12]. It assembles an 80-token sequence (world tokens, action history, learnable chunk queries) and predicts K=5 future actions per frame. Temporal prediction aggregation averages overlapping chunks at inference to remove jitter. Goal directions are computed from pose trajectories; no manual waypoint annotation is required.
- A PointPillars encoder that is small and fast enough that the full stack runs at over 30 FPS on a single GPU, well above the 10 Hz refresh rate of the OS1-64 LiDAR.
- A cross-dataset training paradigm. The world model is pretrained on LidarDustX [13] and GOOSE-3D [14]; the decision transformer is trained on RELLIS-3D. The two sets do not overlap, so the world model is evaluated on terrain it has never seen during pretraining. Test accuracy drops only 0.79% when ground-truth observations are replaced with the world model’s future-frame predictions.
- A focal-loss training recipe with automatic inverse-frequency class weights and label smoothing, the only mitigation we apply for the heavy class imbalance in RELLIS-3D action labels.
- A predictive evaluation that swaps the current LiDAR frame for the world model’s prediction and measures how much downstream action selection changes. Overall, 98.82% of predictions stay the same.

## 2. Related Work

### 2.1. Off-Road Terrain Perception

LiDAR-based terrain perception has been studied extensively for autonomous off-road navigation. Early methods relied on geometric features such as slope, roughness, and step height for terrain classification [3]. More recent approaches employ deep learning on point clouds [15,16] or BEV projections [17,18] to learn terrain representations. The RELLIS-3D dataset [19] provides annotated LiDAR scans across diverse off-road terrain types, enabling supervised learning of terrain semantics.

Traversability estimation is a key component of off-road navigation. Methods range from geometry-based approaches [20] to learning-based frameworks that predict traversability from point clouds or images [1,21]. Self-supervised approaches have shown particular promise, learning traversability from proprioceptive signals during traversal [22,23].

### 2.2. World Models for Robotics

World models learn compact representations of environment dynamics, enabling an agent to predict future states and plan accordingly. Dreamer [7] and DreamerV3 [8] demonstrated the effectiveness of world models for continuous control tasks, learning latent dynamics models that support imagination-based planning. MILE [24] applied world models to urban autonomous driving, jointly predicting future BEV semantic maps and planning trajectories.

In the context of 3D environments, Point Cloud World Models [25] and related approaches have explored predicting future 3D scenes from LiDAR observations. Our work extends this direction to off-road environments, where terrain dynamics are more complex and varied than structured road scenes.

### 2.3. Decision Transformers

The Decision Transformer [9] reformulated offline reinforcement learning as a sequence modeling problem, conditioning action generation on desired returns. Subsequent work has extended this framework with trajectory transformers [26], online fine-tuning [10], and multi-modal conditioning [27]. In the driving domain, transformers have been applied to trajectory prediction [28] and end-to-end planning [29].

Our approach differs from prior decision transformer work in three key aspects: (1) we condition decisions on learned world model representations rather than raw observations, enabling abstraction of terrain features; (2) we adopt action chunking [12] with TemporalEnsemble over a full-sequence token architecture that attends jointly to spatial (BEV world tokens), temporal (action-history tokens), and predictive (chunk query) tokens; and (3) we use discrete navigation actions with automatically derived goal directions suited to off-road settings rather than continuous trajectory waypoints typical of urban driving.

### 2.4. Behavioral Cloning for Navigation

Behavioral cloning (BC) learns policies directly from expert demonstrations [30,31]. While BC suffers from a distribution shift in theory, it remains a practical and effective approach when paired with data augmentation and appropriate action representations [32]. For off-road navigation, BC has been applied using various input modalities, including RGB images [33], LiDAR [5], and multi-modal inputs [34]. Our work employs BC for the decision transformer training phase, using expert trajectories from the RELLIS-3D dataset.

### 2.5. Position of TerrainFormer in the Literature

**Figure 1:**
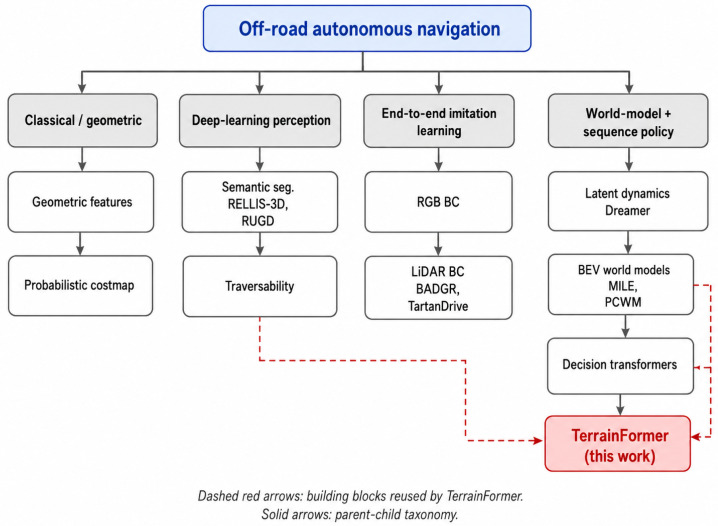
Taxonomy of related work on off-road autonomous navigation. The four solid-line branches are classical geometric approaches, deep learning perception (semantic segmentation and traversability), end-to-end imitation learning, and the more recent world-model-plus-sequence-policy family. TerrainFormer sits at the leaf of the last branch. Dashed red arrows mark the prior components TerrainFormer reuses: BEV world models from urban driving [24,25], decision-transformer architectures from offline RL [9,10,26], and self-supervised traversability/segmentation targets from deep learning perception [1,21,22].

The four lines of work surveyed above are not independent; in practice, they form a layered taxonomy. Figure 1 shows the taxonomy with TerrainFormer’s slot highlighted. Classical terrain analysis and deep learning perception solve subproblems that an end-to-end policy must otherwise learn from scratch, and world models plus decision transformers provide the architectural components that let a single network do both. TerrainFormer takes the BEV-world-model formulation that proved effective in urban driving (MILE, PCWM) and pairs it with a decision-transformer policy head originally developed for offline RL, with three off-road-specific changes: a PointPillars front-end sized for an on-vehicle compute budget, an action-chunked output trained with focal loss to keep minority maneuvers learnable under the heavy class imbalance of off-road driving, and a deliberate cross-dataset training split that lets us evaluate the world model on terrain it never saw during pretraining.

Table 1 and Table 2 compare TerrainFormer against representative systems on the architectural and methodological axes that drive their performance differences. Table 1 reports the architectural backbones (domain, perception, policy); Table 2 reports the deployment-relevant capabilities (action representation, cross-dataset training, real-time inference). The point of the comparison is not to claim TerrainFormer is uniformly better; several of the listed systems were designed for urban driving rather than off-road and use different action representations, sensor stacks, or training datasets. What the tables do show is which design choices are shared, which are specific to TerrainFormer, and where each design choice comes from in the literature.

Three observations from Table 1 and Table 2 are noteworthy. (1) BADGR [33] and TartanDrive [34] are the closest off-road comparators, both end-to-end. Neither separates terrain representation from action selection, so the perception layer cannot be evaluated independently or transferred. TerrainFormer’s two-phase split is what makes the cross-dataset measurement in Section 5 possible at all. (ii) MILE [24] and PCWM [25] bring world-model machinery to urban driving with the same BEV-then-transformer pattern we use, but they consume a single urban dataset and output continuous trajectories. The architectural lineage is real, but the off-road action vocabulary and cross-dataset protocol are new here. (3) The original Decision Transformer [9] treats policy learning as autoregressive sequence modeling but operates on state vectors, not perception. Pairing it with a BEV world model is the architectural step that turns it into a perception-aware policy.

## 3. Methodology

TerrainFormer comprises four principal components arranged in a hierarchical pipeline: (1) a LiDAR encoder that processes raw 3D point clouds into dense features, (2) a BEV projection module that creates spatial feature maps suitable for downstream processing, (3) a world model that learns terrain dynamics and latent representations through self-supervised pretraining, and (4) a temporal decision transformer that predicts discrete navigation actions conditioned on temporal context and automatically derived goal directions. Training proceeds in two phases: self-supervised world model pretraining (Phase 1) using multi-dataset LiDAR observations, followed by behavioral cloning for the temporal decision transformer (Phase 2) on separate sequences.

**Figure 2:**
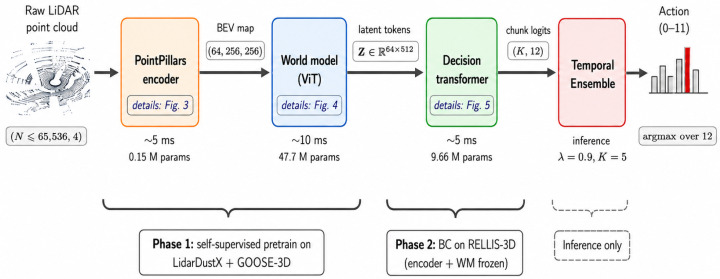
High-level dataflow of TerrainFormer. A raw LiDAR point cloud (left) passes through four stages: the PointPillars encoder produces a 64×256×256 BEV feature map, the ViT-based world model compresses it to 64 latent tokens $Z\in R^{64\times 512}$, the decision transformer produces (K,12) chunk logits, and TemporalEnsemble aggregates overlapping chunks across the last K=5 frames into a single discrete action. Component internals are deliberately omitted here and are expanded in Figure 3 (encoder), Figure 4 (world model), and Figure 5 (decision transformer). Per-stage latency and parameter counts are annotated below each block; total end-to-end inference is ∼20 ms (∼50 FPS) on a NVIDIA Quadro RTX 8000 GPU (NVIDIA Corporation, Santa Clara, CA, USA). Bottom braces show the two-phase training schedule: encoder + world model pretrained self-supervised on LidarDustX + GOOSE-3D (Phase 1, blue), then frozen while the decision transformer is trained with behavioral cloning on RELLIS-3D (Phase 2, green).

**Figure 3:**
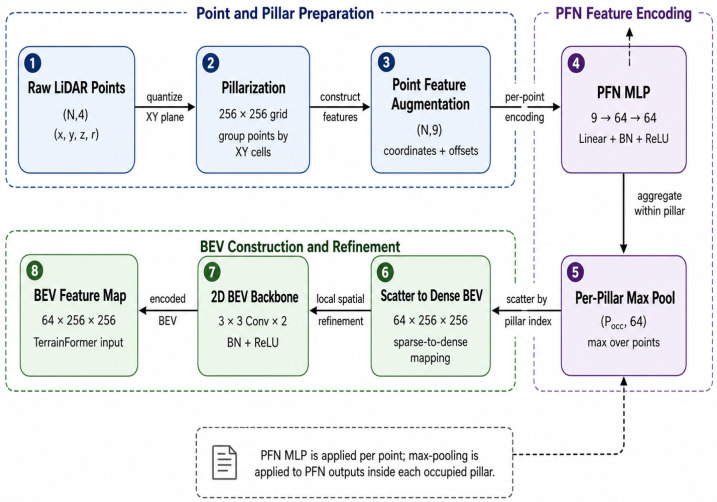
Component view of the PointPillars LiDAR encoder used by TerrainFormer. The pipeline is read left-to-right (top row) then folded back right-to-left (bottom row). Critically, the per-point MLP operates independently on every point and is not pooled itself; max-pooling is applied to the MLP outputs within each pillar (Section 3.1). Tensor shapes are annotated below each block.

**Figure 4:**
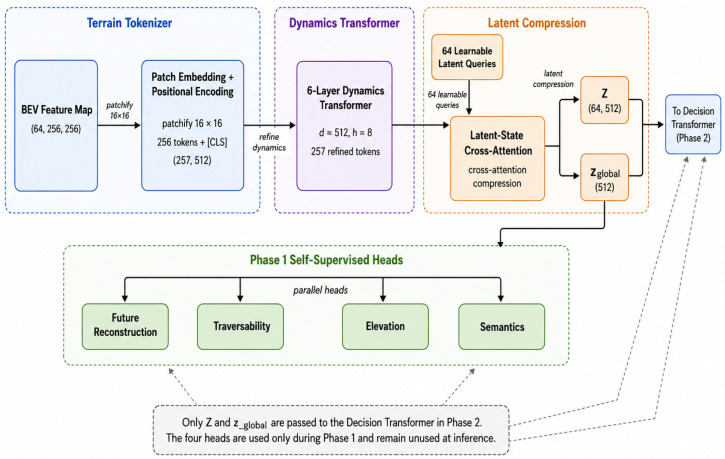
Component view of the world model. The three internal modules (terrain tokenizer, dynamics transformer, and latent compression) and the four self-supervised heads used during Phase 1 pretraining are shown explicitly. Only Z (64 latent tokens) and $z_{\mathrm{global}}$ are passed on to the decision transformer in Phase 2; the four prediction heads remain unused at inference, and after Phase 1, the entire world model is frozen.

**Figure 5:**
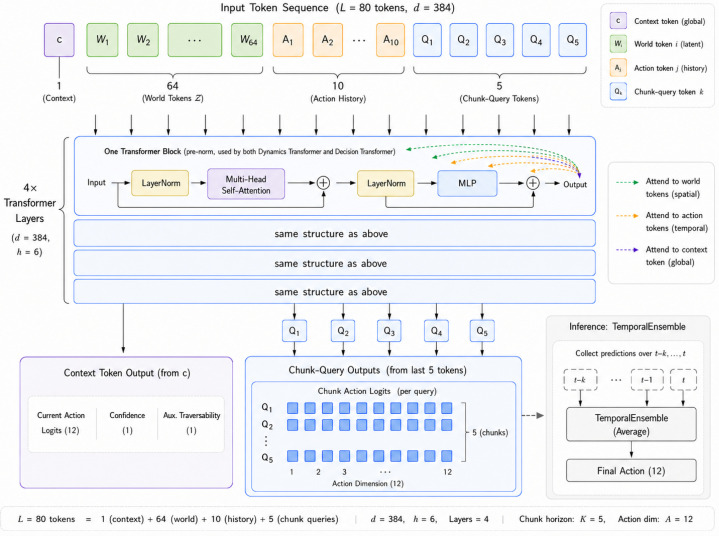
Component view of the decision transformer. The 80-token sequence (top) is composed of one context token c, the 64 world latent tokens, 10 action-history tokens, and 5 learnable chunk-query tokens. Dashed red arrows from one chunk query illustrate the cross-modal attention pattern: every chunk query attends to all spatial (world) and temporal (action-history) tokens simultaneously. The context-token output predicts the current action and confidence; the last five output positions predict the action chunk (B,K=5,A=12); temporal prediction aggregation at inference aggregates overlapping chunks with exponential-decay weighting.

Figure 2 gives the high-level data flow at a glance: a point cloud (top) is encoded into a BEV feature map, compressed by the world model into 64 latent tokens, and finally consumed by the decision transformer to produce action chunks. To keep the high-level view readable, Figure 2 deliberately omits internal block details; expanded views of each component are provided in subsequent figures referenced from the relevant subsections: the PointPillars encoder in Figure 3 (Section 3.1), the world-model internals in Figure 4 (Section 3.2), and the decision-transformer token layout in Figure 5 (Section 3.3). The remainder of this section walks through the components in pipeline order, with each subsection describing one block of Figure 2.

### 3.1. LiDAR Encoder

The LiDAR encoder processes raw 3D point clouds of up to N=65,536 points, where each point has four features (x,y,z,i) representing spatial coordinates and intensity. We employ a PointPillars-based encoder [18] that directly converts point clouds to BEV features, chosen for its architectural alignment with our BEV-centric pipeline and real-time performance characteristics. Figure 3 shows the encoder in isolation, with each pipeline stage labelled.

#### 3.1.1. PointPillars Architecture

The encoder operates as follows:

- Pillarization: Points are discretized into vertical pillars on a 256×256 BEV grid covering 100m×100m (x,y∈[−50,50] m). Each pillar aggregates up to 32 points; pillars exceeding this cap are uniformly subsampled.
- Feature Augmentation: Each point is augmented with 5 additional features: $\left(x_{c},y_{c},z_{c}\right)$ offset from pillar centroid and $\left(x_{p},y_{p}\right)$ offset from pillar geometric center, yielding a 9-dimensional per-point input.
- Pillar Feature Network: A two-layer pointwise MLP (9→64→64 with BatchNorm and ReLU) is applied independently to every point. The MLP itself is not pooled; rather, the per-point 64-dimensional outputs are subsequently aggregated within each pillar by an element-wise max operation (implemented via vectorized scatter_reduce). The result is a single 64-dimensional feature per occupied pillar, which is then scattered back onto the 256×256 BEV grid (empty cells set to zero).
- BEV Backbone: Two 3×3 convolutional layers with BatchNorm and ReLU refine the resulting 64×256×256 spatial feature map.

The PointPillars encoder achieves ∼5 ms latency on a single NVIDIA Quadro RTX 8000 GPU (NVIDIA Corporation, Santa Clara, CA, USA) (All latency numbers in this paper were measured on the same workstation, which was equipped with an NVIDIA Quadro RTX 8000 GPU (NVIDIA Corporation, Santa Clara, CA, USA; 48 GB GDDR6 VRAM, Turing TU102 architecture), an Intel Core i9 CPU, and 64 GB RAM, running PyTorch 2.1 with FP16 inference. Each value is the mean over 1000 inference passes after a 100-pass warm-up; standard deviations were below 0.4 ms in all cases), enabling real-time inference at 10 Hz LiDAR rates with ample margin for downstream processing.

#### 3.1.2. Encoder Design Rationale

We considered alternative architectures, including PointNet++ [16], which offers hierarchical set abstraction for fine-grained local geometry learning. However, PointPillars was selected for three reasons: (1) architectural alignment—PointPillars produces BEV features directly, matching the world model’s expected input without intermediate projection layers; (2) latency—PointPillars achieves 5× lower encoder latency, critical for real-time navigation at 10 Hz; (3) compensating capacity—the 6-layer Dynamics Transformer provides sufficient representational capacity to learn fine-grained terrain patterns that hierarchical point encoders would capture at the encoder level.

Table 3 compares the two encoder architectures, including per-component FPS. PointPillars achieves ∼200 FPS at the encoder stage (vs. ∼40 FPS for PointNet++), and the full TerrainFormer pipeline runs at ∼50 FPS end-to-end. A hypothetical PointNet++ variant would achieve only ∼25 FPS end-to-end, falling below the 30 FPS threshold required for responsive real-time navigation and exceeding the 100 ms frame budget when combined with downstream processing.

The 5× latency advantage of PointPillars (∼5 ms vs. ∼25 ms) arises from three distinct architectural factors, not simply from parameter count (PointPillars has ∼10× fewer parameters, but parameter count alone does not explain the wall-clock gap):

- Memory layout (dominant factor): PointPillars produces a regular, dense 256×256 BEV tensor in a single scatter_reduce call, which maps directly onto GPU memory-coalescing hardware. PointNet++ instead performs iterative neighborhood queries (ball_query, KNN) on irregular point sets; these are scattered memory accesses with low cache-line utilization and high index-arithmetic overhead.
- Hierarchy depth: PointNet++ stacks 3–4 set-abstraction layers, each requiring its own grouping operation. PointPillars groups points exactly once (during pillarization) and runs the remaining layers as standard 2D convolutions, for which cuDNN already has fast kernels.
- Output-format alignment: PointPillars produces BEV features natively; the downstream ViT world model can consume them without an intermediate projection step. A PointNet++ variant would require an additional point-to-BEV rasterization layer (~2 ms in our profiling), which the table does not separately charge to its column.

These factors compound: even with TensorRT optimization, PointNet++ remains ∼3× slower than PointPillars in our profiling. Empirically, for BEV-based downstream tasks, PointPillars consistently matches PointNet++ performance on established benchmarks while enabling TensorRT optimization for embedded deployment, which is critical for the real-time off-road navigation setting where rapid terrain changes and dynamic obstacles demand a low-latency perception-to-action pipeline.

#### 3.1.3. BEV Output

The encoder produces a BEV feature map $F_{\mathrm{BEV}}\in R^{64\times 256\times 256}$ and a global feature vector $z_{\mathrm{encoder}}\in R^{1024}$ via adaptive pooling followed by linear projection.

### 3.2. World Model

The world model learns to predict terrain dynamics and extract rich representations from BEV features through self-supervised pretraining. It consists of three components: a terrain tokenizer, a dynamics transformer, and a latent state module. Figure 4 shows the three components in sequence together with the four self-supervised prediction heads.

#### 3.2.1. Terrain Tokenizer

The BEV feature map $F_{\mathrm{BEV}}$ is divided into non-overlapping patches of size 16×16 pixels, yielding $P={\left(256/16\right)}^{2}=256$ patches. Each patch is projected to a token of dimension d=512 via a convolutional embedding layer. Learned positional encodings $E_{\mathrm{pos}}\in R^{256\times 512}$ and a learnable [CLS] token are prepended, producing an input sequence of 257 tokens.

#### 3.2.2. Dynamics Transformer

The token sequence is processed by a 6-layer transformer encoder with the following configuration per layer: $d_{\mathrm{model}}=512$, h=8 attention heads, head dimension $d_{k}=64$, MLP ratio 4× (intermediate dimension 2048), and dropout rate 0.1. The architecture uses pre-norm (LayerNorm before attention and MLP), following the convention of Vision Transformers [35].

#### 3.2.3. Latent State Compression

To produce a compact representation for the decision transformer, we employ a cross-attention-based compression module. A set of 64 learnable latent queries $Q_{\mathrm{latent}}\in R^{64\times 512}$ attend to the 257 transformer output tokens via multi-head cross-attention (h=8 heads), producing compressed latent tokens $Z\in R^{64\times 512}$. A global feature vector $z_{\mathrm{global}}\in R^{512}$ is obtained by mean pooling over the latent tokens, followed by a linear projection.

#### 3.2.4. Prediction Heads

Four parallel prediction heads decode the latent representations for self-supervised training:

- Future Reconstruction: Predicts the next 5 frames’ BEV features at patch resolution, output $R^{5\times 64\times 16\times 16}$.
- Traversability: Transposed convolution upsampling (4×) to produce a binary traversability map $R^{1\times 256\times 256}$ with sigmoid activation.
- Elevation: Same architecture, predicting continuous elevation values $R^{1\times 256\times 256}$.
- Semantics: Transposed convolution upsampling to produce per-class logits $R^{C\times 256\times 256}$ where C=20 for RELLIS-3D semantic classes.

### 3.3. Decision Transformer

The decision transformer predicts discrete navigation actions using a token-sequence architecture that provides full cross-attention between spatial world features, temporal action history, and learnable future-action query tokens. The token layout and output heads are illustrated in Figure 5.

#### 3.3.1. Token Sequence Architecture

Inspired by action chunking [12], the model simultaneously predicts the next K=5 actions per frame rather than a single step, improving temporal coherence and enabling anticipatory look-ahead. The transformer processes a fixed-length sequence of L=80 tokens:

(1) $$X=\left[c;\;W_{1:64};\;A_{1:10};\;Q_{1:5}\right]\;\in R^{80\times 384}$$

where $c\in R^{384}$ is a fused context token, $W_{1:64}$ are projected world latent tokens (BEV spatial features), $A_{1:10}$ are per-step action history embeddings (temporal features), and $Q_{1:5}$ are *K* learnable chunk query tokens that represent the *K* future action predictions.

Learnable positional embeddings $E_{\mathrm{pos}}\in R^{80\times 384}$ are added to the full sequence to encode token order.

#### 3.3.2. Context Aggregator

The context token c fuses four information sources through a two-layer MLP followed by cross-attention over the world latent tokens Z:

- World global feature: $z_{\mathrm{global}}\in R^{512}$, linearly projected to $R^{384}$.
- Vehicle state: $s\in R^{6}$ encoding velocity $\left(v_{x},v_{y}\right)$, angular velocity ω, and orientation (pitch, roll, yaw).
- Goal direction: $g\in R^{2}$ (Section 3.5).
- Action aggregate: $e_{a}\in R^{128}$, mean-pooled over 10 per-step action embeddings.

(2) $$c=\mathrm{CrossAttn}\;\left({\mathrm{MLP}}_{\mathrm{fuse}}\left(\left[\mathrm{proj}\left(z_{\mathrm{global}}\right);e_{s};e_{g};e_{a}\right]\right),\;Z\right)\in R^{384}$$

#### 3.3.3. Token Projections

World latent tokens $Z\in R^{64\times 512}$ are projected to $R^{64\times 384}$ via a linear layer. Action history embeddings are produced per-step (preserving temporal structure) and projected to $R^{10\times 384}$ via a separate linear layer. Chunk queries $Q\in R^{5\times 384}$ are learnable parameters initialized with a truncated-normal distribution (σ=0.02).

#### 3.3.4. Transformer Backbone

The 80-token sequence is processed by a 4-layer transformer with $d_{\mathrm{model}}\;=\;384$, h=6 attention heads, head dimension $d_{k}\;=\;64$, MLP ratio 4×, and dropout 0.1, using pre-norm (LayerNorm before attention and MLP). Full self-attention over all 80 tokens enables each chunk query to attend simultaneously to BEV spatial context (world tokens) and temporal action history (action tokens), providing richer conditioning than single-token architectures.

#### 3.3.5. Output Heads

- Current-step action: The context token output $x_{0}\in R^{384}$ is decoded by an action head (MLP 384→12), a confidence head (MLP 384→1 with sigmoid), and an auxiliary traversability head (MLP 384→1 with sigmoid).
- Action chunk: Each of the last K=5 output tokens $x_{-5:}\in R^{5\times 384}$ is independently decoded by the shared action head, yielding chunk logits $\in R^{K\times 12}$.

#### 3.3.6. Temporal Ensembling at Inference

During inference, each frame produces a K=5 action chunk. Overlapping chunks from the last *K* consecutive frames are aggregated using Temporal Ensemble [12] with exponential-decay weighting:

(3) $${\hat{a}}_{t}=\arg\max_{a}\;\frac{\sum_{k=0}^{K-1}\lambda^{k}\cdot {\mathrm{chunk}}_{t-k}\left[k\right]}{\sum_{k=0}^{K-1}\lambda^{k}},\;\lambda =0.9$$

where ${\mathrm{chunk}}_{t-k}\left[k\right]$ is the *k*-th step prediction from the chunk generated *k* frames ago. More recent predictions receive higher weight. This averaging suppresses prediction jitter and smooths action transitions without latency overhead.

### 3.4. Action Space

We define a discrete action space of 12 forward-motion actions that combine speed and steering commands, derived from continuous vehicle poses via velocity and turn-rate thresholding (Table 4). The action space is designed specifically for forward-only driving scenarios characteristic of the RELLIS-3D dataset, which contains no backward motion. Actions are classified using a turn-first approach: the turn-rate magnitude determines the action category, with forward-speed actions reserved for minimal-turn scenarios.

Action labels are derived from ground-truth pose sequences by computing linear velocity and turn rate between consecutive frames (Δt=0.1 s). Using a turn-first classification approach, turn rate magnitude is evaluated first: sharp (|ω|≥0.6 rad/s), medium (0.3–0.6), slight (0.1–0.3), or minimal (<0.1). Turn actions (4–9) are assigned regardless of velocity; forward-speed actions (0–3) apply only when |ω|<0.1. Combined actions (10–11) represent fast forward motion (v≥1.0 m/s) with sharp turning. Since RELLIS-3D contains exclusively forward driving trajectories (no backward motion), the action space excludes backward-related actions, simplifying the classification task while maintaining expressiveness for off-road navigation.

### 3.5. Goal Direction Computation

A critical component of directionally conditioned navigation is the goal direction input, which informs the model where the vehicle should navigate. Rather than relying on externally provided waypoints, we compute goal directions automatically from the vehicle’s future trajectory encoded in the pose sequence.

For each frame *t*, the goal direction $g_{t}\in R^{2}$ is computed as the normalized displacement vector from the current position to a future position (lookahead k=5 frames), expressed in the vehicle’s local coordinate frame:

(4) $$d_{t}=p_{t+k}-p_{t}$$

(5) $$g_{t}=R\left(-\psi_{t}\right)\cdot \frac{d_{t}}{∥d_{t}∥}$$

where $p_{t}=\left(x_{t},y_{t}\right)$ is the vehicle position at frame *t*, $\psi_{t}$ is the yaw angle extracted from the rotation matrix, and $R(-\psi_{t})$ is the 2D rotation matrix that transforms world coordinates to the vehicle’s local frame. This transformation ensures that $g_{t}=\left(g_{x},g_{y}\right)$ represents the goal direction relative to the vehicle’s heading:

- $g_{x}>0$: goal is ahead of the vehicle
- $g_{y}>0$: goal is to the left of the vehicle
- $g_{y}<0$: goal is to the right of the vehicle

This automatic goal derivation provides several advantages: (1) it ensures consistency between goal direction and actual vehicle behavior during training, (2) it enables the model to learn turn-direction associations (left turns correspond to positive $g_{y}$), and (3) during inference, goal directions can be provided manually or computed from planned trajectories.

- Pose source. The current pose $\left(p_{t},\psi_{t}\right)$ used in Equations (2) and (3) is not an output of TerrainFormer; it is supplied by an upstream module that is assumed to be available on the target platform. During training, poses are read directly from the RELLIS-3D ground-truth pose file (poses.txt), which provides a 3×4[R|t] transform per frame from a LiDAR-inertial SLAM solution. During inference, TerrainFormer expects the host platform to provide pose from any standard source: LiDAR odometry (e.g., LOAM, LIO-SAM), wheel odometry combined with an IMU and an extended Kalman filter, or GNSS-INS. The system only requires relative pose accuracy over a 5-frame (0.5 s) horizon for goal computation; absolute global accuracy is not required. The future position $p_{t+k}$ used during training comes from the same pose log; at inference time, it is replaced by either (a) a user-specified waypoint, (b) a planner-provided sub-goal, or (c) a constant “go straight” default ($g_{t}={\left(1,0\right)}^{⊤}$) when no goal source is available.

### 3.6. Training Pipeline

#### 3.6.1. Phase 1: World Model Pretraining Loss

The world model (LiDAR encoder + BEV projection + world model) is trained via self-supervised learning on multiple LiDAR datasets. The combined loss is as follows:

(6) $$L_{\mathrm{WM}}=\lambda_{r}L_{\mathrm{recon}}+\lambda_{t}L_{\mathrm{trav}}+\lambda_{e}L_{\mathrm{elev}}+\lambda_{s}L_{\mathrm{sem}}+\lambda_{c}L_{\mathrm{con}}$$

where $L_{\mathrm{recon}}$ is the Chamfer distance for future-frame reconstruction ($\lambda_{r}=1.0$), $L_{\mathrm{trav}}$ is the binary cross-entropy for traversability ($\lambda_{t}=0.5$), $L_{\mathrm{elev}}$ is mean squared error for elevation ($\lambda_{e}=0.3$), $L_{\mathrm{sem}}$ is the cross-entropy for semantic segmentation ($\lambda_{s}=0.5$), and $L_{\mathrm{con}}$ is the contrastive loss with temperature τ=0.07 ($\lambda_{c}=0.2$).

#### 3.6.2. Phase 2: Decision Transformer Training Loss

The decision transformer is trained via behavioral cloning with the PointPillars encoder and world model parameters frozen. To address severe class imbalance, we employ focal loss [36] with automatic inverse-frequency class weighting:

(7) $$L_{\mathrm{focal}}=-\alpha_{c}{\left(1-p_{t}\right)}^{\gamma }\log\left(p_{t}\right)$$

where $p_{t}$ is the predicted probability for the true class, γ=2.0 is the focusing parameter, and $\alpha_{c}$ is the class-specific weight:

(8) $$\alpha_{c}=\frac{N_{\mathrm{total}}}{C\cdot N_{c}},\;C=12$$

Label smoothing ϵ=0.1 is applied to prevent overconfident predictions.

- Action chunking loss. For each training frame, the dataset provides K=5 future ground-truth action labels $\left\{a_{t},a_{t+1},\ldots ,a_{t+K-1}\right\}$. The chunk head is supervised by applying the same focal loss to all *K* steps jointly:
  (9) $$L_{\mathrm{chunk}}=L_{\mathrm{focal}}\;\left({\hat{L}}_{\left(B\cdot K,12\right)}^{\mathrm{chunk}},\;a_{\left(B\cdot K\right)}\right)$$
  where chunk logits (B,K,12) are reshaped to (B·K,12) before loss computation.
- Auxiliary traversability loss. The traversability head is supervised using the mean value of the BEV traversability map, computed geometrically from the point cloud when ground-truth labels are unavailable (Section 3).

The combined Phase 2 loss is as follows:

(10) $$L_{\mathrm{DT}}=L_{\mathrm{focal}}+0.2\;L_{\mathrm{trav}}+0.5\;L_{\mathrm{chunk}}$$

The 0.5 chunk weight balances look-ahead supervision against the primary current-step objective without destabilizing early training.

## 4. Experimental Setup

### 4.1. Datasets

**Figure 6:**
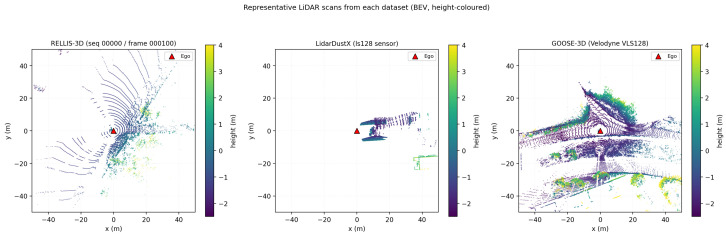
Representative LiDAR scans from each dataset, projected to BEV and colored by height (clip range −2.5 m to +4.0 m). From left to right: RELLIS-3D—vegetation-heavy off-road, Ouster OS1-64 (∼37 k points/scan); LidarDustX—dusty construction environment, multi-sensor capture (∼111 k points/scan with the LS128 sensor shown); and GOOSE-3D—mixed European outdoor (forests, fields, campus), Velodyne VLS128 (∼199 k points/scan). The red triangle marks the ego vehicle.

We use three publicly available off-road LiDAR datasets that play complementary roles: RELLIS-3D for action-conditioned decision training, and LidarDustX plus GOOSE-3D for self-supervised world-model pretraining on terrain types the decision transformer never sees during its own training. The three datasets are described individually below; the resulting two-phase training assignment is summarized in Section 4.1.4. Representative LiDAR scans from each dataset are shown in Figure 6.

#### 4.1.1. RELLIS-3D

RELLIS-3D [19] is a multi-terrain off-road dataset collected using a Clearpath Warthog robot (Clearpath Robotics Inc., Kitchener, ON, Canada) equipped with an Ouster OS1-64 LiDAR sensor (Ouster, Inc., San Francisco, CA, USA) (64 channels, 120 m range, 10 Hz). The dataset contains 5 sequences (00000–00004) recorded across diverse off-road terrains, including grass, mud, puddle, dirt, and forest environments. Each LiDAR frame contains up to 65,536 points with (x,y,z,intensity) features. Semantic annotations cover 20 terrain classes (grass, tree, pole, water, vehicle, asphalt, mud, gravel, etc.). Ground-truth 6-DoF poses are provided via SLAM, which we use for both action-label derivation and goal-direction computation (Section 3.5).

#### 4.1.2. LidarDustX

LidarDustX [13] is a multi-sensor LiDAR dataset captured in dusty off-road construction environments. It contains 7562 frames across 6 different LiDAR sensors: LS128 (629 frames), LS64 (1116 frames), LY150 (410 frames), LY300 (671 frames), M1 (3112 frames), and Ouster (1624 frames). Each sensor captures frames across multiple recording sequences (174 sequences total). The dataset provides per-point semantic labels (11 classes: background, dust, ground, mound, stone, obstacle, engineering, car, truck, trailer, pedestrian) but does not include vehicle pose or odometry data.

Since no action labels are available, LidarDustX is used exclusively for Phase 1 (world model pretraining), where it provides diverse multi-sensor LiDAR observations across dusty and degraded visual conditions. Sensor-specific preprocessing handles NaN values in M1 sensor data and normalizes Ouster intensity from [0,2701] to [0,255]. A 70/15/15 chronological split is applied per sequence, yielding 5294 training, 1134 validation, and 1134 test frames.

#### 4.1.3. GOOSE-3D

GOOSE-3D [14] is a large-scale outdoor semantic segmentation dataset captured using a Velodyne VLS128 LiDAR sensor (Velodyne Lidar, Inc., San Jose, CA, USA; 128 channels) across 23 diverse outdoor scenes in Germany, spanning forests, fields, campus environments, rural roads, and varying weather conditions, including rain and snow. The dataset contains 7719 annotated LiDAR frames with an average of approximately 186,000 points per frame—significantly denser than both RELLIS-3D and LidarDustX. Per-point semantic annotations cover 64 classes, including terrain surfaces (asphalt, gravel, soil, grass, cobble), vegetation (forest, bush, tree crown, tree trunk, hedge), structures (building, wall, fence, bridge), and dynamic objects (car, person, bicycle, truck). Labels are stored as uint32 values encoding both semantic class (label∧0xFF) and instance ID (label≫8).

No vehicle pose or odometry data is available, restricting GOOSE-3D to Phase 1 (world model pretraining). Frames are temporally sparse (10–200 s between consecutive frames), so temporal history and future chains are often unavailable; GOOSE-3D primarily contributes to single-frame prediction tasks (traversability, elevation, and semantic segmentation). A 70/15/15 chronological split is applied per scene, yielding approximately 5403 training, 1158 validation, and 1158 test frames.

#### 4.1.4. Cross-Dataset Training Paradigm

Having introduced the three datasets above, we now describe how they are assigned across the two training phases. To rigorously evaluate the generalization of learned terrain representations, we enforce a strict zero-overlap policy: the world model never sees any RELLIS-3D frame during pretraining, and the decision transformer never sees any LidarDustX or GOOSE-3D frame during behavioral cloning. Table 5 summarizes the assignment.

This separation ensures that the decision transformer is evaluated on terrain that the world model has never observed during pretraining, providing a rigorous test of cross-domain generalization. Action labels and goal directions are derived from consecutive pose pairs in the RELLIS-3D ground-truth pose log by computing linear velocity, yaw rate, and 5-frame future displacement, then discretizing into 12 forward-motion action classes (Section 3.4) and 2D goal vectors (Section 3.5). During decision training, each input point cloud is first passed through the frozen pretrained world model to obtain BEV-encoded latent features, which, together with the auxiliary inputs (state vector, goal direction, action history), are processed by the temporal decision transformer to predict actions.

### 4.2. Data Preprocessing

Point clouds are filtered to retain points within a range of [0.5,70.0] m from the sensor origin. Voxel downsampling with a voxel size of 0.1 m reduces point density while preserving geometric detail. Coordinates are centered on the unmanned ground vehicle (UGV), and intensity values are normalized to [0,1].

### 4.3. Data Augmentation

To mitigate overfitting to sequential LiDAR data, in which consecutive frames exhibit high similarity, we apply the following augmentations during training only:

- Random rotation: $\pm 45^{\circ }$ around the vertical (*z*) axis.
- Random horizontal flip: 50% probability along the *x*-axis.
- Random scale: uniform in [0.95,1.05].
- Random translation: uniform in [−0.5,0.5] m per axis.
- Gaussian point noise: σ=0.02.
- Intensity jitter: uniform in [−0.1,0.1].

### 4.4. Training Details

#### 4.4.1. Phase 1: World Model Pretraining

The world model (47.70 M parameters) is trained for 100 epochs using AdamW [37] with a learning rate of $1\times 10^{-4}$, a weight decay of 0.01, and β=(0.9,0.999). The batch size is 16 with gradient accumulation over 2 steps (effective batch size 32). A cosine learning rate schedule with 5 warm-up epochs decays the learning rate to a minimum of $1\times 10^{-6}$. Gradient norms are clipped at 1.0. Mixed-precision (FP16) training is used throughout.

Critically, the world model is trained only on LidarDustX (5229 frames) and GOOSE-3D (5403 frames), totaling 10,632 training samples. RELLIS-3D is excluded entirely to enable cross-dataset evaluation.

#### 4.4.2. Phase 2: Decision Transformer Training

The temporal decision transformer (9.66 M parameters) is trained for 50 epochs with the LiDAR encoder and world model frozen. We use AdamW with a learning rate of $3\times 10^{-4}$, a weight decay of 0.05, and β=(0.9,0.95). The batch size is 32 with no gradient accumulation. A cosine schedule with 3 warm-up epochs and a minimum learning rate of $1\times 10^{-6}$ is employed.

- Class Imbalance Mitigation: Focal loss with γ=2.0 and automatic inverse-frequency class weights address class imbalance in the action distribution. Label smoothing with ϵ=0.1 prevents overconfident predictions. Recovery scenarios are oversampled with a weight of 2.0, and action balancing is enabled during batch construction.
- Goal Direction Training: Goal directions are computed from pose trajectories and fed in as an input at training time, so the policy learns to condition on direction without needing waypoint annotation. A 5-frame lookahead is sufficient enough to capture turn intent at 10 Hz and short enough that pose noise has not propagated.

All five RELLIS-3D sequences (00000–00004) contribute to Phase 2 under the 70/15/15 chronological split described in Section 4.1.4 (approximately 3640 train/780 val/780 test frames; test contains 2033 chunk-aligned samples). This is the first time TerrainFormer encounters RELLIS-3D in any form, because Phase 1 was trained exclusively on LidarDustX and GOOSE-3D—the world model has never observed RELLIS terrain before Phase 2 begins.

#### 4.4.3. Hardware and Reproducibility

All experiments were conducted on a single NVIDIA Quadro RTX 8000 (48 GB GDDR6 VRAM, Turing TU102 architecture) with CUDA 11.8 and PyTorch 2.1. Phase 1 training took approximately 13 h; Phase 2 took approximately 5 h. Random seeds were fixed (seed = 42) for Python 3.10, NumPy 1.26.4, and PyTorch 2.1.0, and torch.backends.cudnn.deterministic = True was set for the Phase 2 evaluation runs. Due to the computational cost of Phase 1 pretraining, we report results from a single training run for each phase rather than from multi-seed runs; this is a limitation of the present study and is noted in Section 7.6. The complete training and evaluation pipeline, including all configuration files and pretrained checkpoints, is available in the project repository to facilitate reproduction.

#### 4.4.4. Training Curves

**Figure 7:**
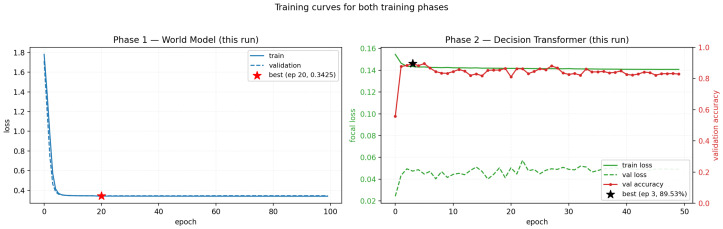
Training curves for both phases. Phase 1 (**left**): world-model training and validation total loss vs. epoch (best validation loss 0.3425 at epoch 20, red star). Phase 2 (**right**): decision-transformer focal loss (green, left axis) and validation accuracy (red, right axis) vs. epoch (best validation accuracy 0.8953 at epoch 3, black star). Solid lines show training metrics; dashed lines show validation metrics. Each curve is from a single training run on a Quadro RTX 8000.

Validation loss as a function of epoch is plotted in Figure 7 for both training phases. The Phase 1 world-model loss decreases monotonically and converges around epoch 20 (best validation loss, 0.3423), after which further epochs yield only marginal improvement. The Phase 2 decision transformer converges very rapidly: with the encoder and world model frozen and supplying strong terrain features, the best validation accuracy of 90.03% is reached at epoch 1 and is not subsequently exceeded over the remaining 49 epochs. Training loss continues to decrease slowly while validation accuracy stays at the same level, indicating that the learned terrain representations from Phase 1 already capture the information needed for action prediction and that the decision transformer mainly has to learn a thin mapping from those features to the 12-class action vocabulary.

### 4.5. Evaluation Protocol

#### 4.5.1. Standard Evaluation

We evaluate action prediction accuracy, macro-averaged precision, recall, and F1 score on the separated test split. Per-class metrics are reported to assess performance across the imbalanced action distribution.

- Metric definitions. For each action class *c*, let ${\mathrm{TP}}_{c}$, ${\mathrm{FP}}_{c}$, ${\mathrm{FN}}_{c}$ denote true positives, false positives, and false negatives, respectively.
  (11) $${\mathrm{Precision}}_{c}=\frac{{\mathrm{TP}}_{c}}{{\mathrm{TP}}_{c}+{\mathrm{FP}}_{c}},\;{\mathrm{Recall}}_{c}=\frac{{\mathrm{TP}}_{c}}{{\mathrm{TP}}_{c}+{\mathrm{FN}}_{c}}$$
- The F1 score is the harmonic mean of precision and recall, penalizing any class where either metric is low:
  (12) $$F1_{c}=\frac{2\cdot {\mathrm{Precision}}_{c}\cdot {\mathrm{Recall}}_{c}}{{\mathrm{Precision}}_{c}+{\mathrm{Recall}}_{c}}$$
- Macro F1 averages $F1_{c}$ equally across all C=12 classes, giving identical weight to rare and frequent actions:
  (13) $${\bar{F1}}_{\mathrm{macro}}=\frac{1}{C}\sum_{c=1}^{C}F1_{c}$$

This is stricter than accuracy (dominated by majority classes) and stricter than weighted F1 (which discounts minority classes by frequency).

#### 4.5.2. PredictiveEvaluation Protocol

To evaluate the quality of the world model’s learned representations for decision-making, we compare two conditions:

- Current frame: The decision transformer receives world model features computed from the ground-truth current LiDAR observation.
- Predicted future frame: The decision transformer receives world model features from the world model’s prediction of the future frame (i.e., the world model predicts what the next observation will look like, and the decision transformer acts on that prediction).

The accuracy difference between these conditions quantifies how much the world model’s prediction errors degrade downstream action selection. We also report the prediction agreement rate: the fraction of test samples where both conditions produce the same action.

## 5. Results

### 5.1. World Model Pretraining

The world model converges to a best validation loss of 0.3423 at epoch 20 during Phase 1 pretraining. This combined loss reflects performance across all four prediction tasks (future reconstruction, traversability, elevation, and semantic segmentation). Critically, the model is trained only on LidarDustX and GOOSE-3D, with no exposure to RELLIS-3D data.

### 5.2. Cross-DatasetGeneralization Results

A key finding is that the world model trained on LidarDustX and GOOSE-3D successfully generalizes to RELLIS-3D for decision-making, despite never having seen RELLIS-3D terrain during pretraining. This validates that the learned terrain representations capture generalizable features (traversability, elevation, obstacles) rather than dataset-specific patterns.

### 5.3. Decision Transformer Performance

#### 5.3.1. Overall Metrics

Table 6 summarizes the decision transformer’s performance on RELLIS-3D, using world model features from a model that was never trained on RELLIS-3D.

The model achieves 87.31% test accuracy despite cross-dataset generalization, with a macro F1 of 0.7948 showing strong balanced performance across all 12 action classes. The two dominant classes (stop 33.3%, fwd_fast 34.8%) account for 68.1% of test samples, yet focal loss and action-chunk supervision prevent the model from collapsing to majority-class predictions.

#### 5.3.2. Per-Class Analysis

Table 7 presents per-class metrics and the action distribution in the test set.

**Figure 8:**
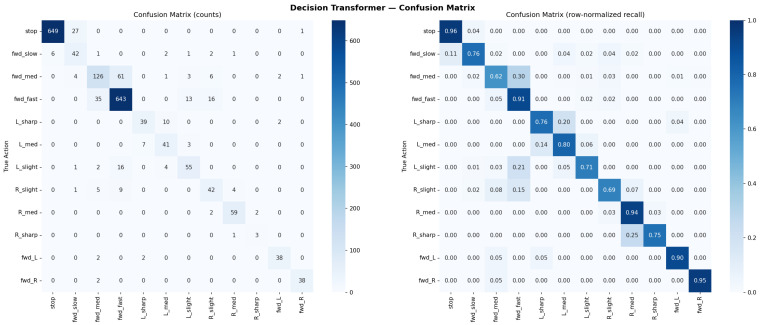
Confusion matrix of the decision transformer on the RELLIS-3D test set. **Left**: raw counts; **Right**: row-normalized per-class recall. Dominant classes (stop, fwd_fast) achieve high diagonal values; all classes show strong diagonal recall.

**Figure 9:**
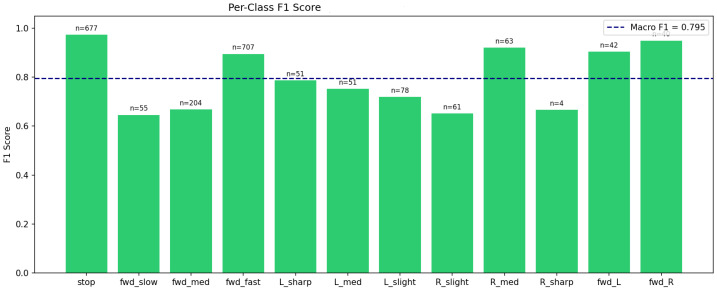
Per-class F1 score with sample support counts. The dashed line shows the macro-averaged F1.

The dominant classes are stop (33.3%) and forward-fast (34.8%), together comprising 68.1% of the test set. Figure 8 and Figure 9 show the confusion matrix and per-class F1 distribution, respectively. Figure 9 shows that all 12 classes achieve F1 ≥0.65, with only the rare R_slight class (61 samples) falling below 0.70. Action chunking and focal loss effectively prevent the majority-class collapse despite the imbalanced distribution.

#### 5.3.3. Confusion Matrix Analysis

The row-normalized confusion matrix reveals several interpretable patterns:

- Strong diagonal for dominant classes: Stop (F1 0.975) and forward-fast (F1 0.896) show near-perfect recall, confirming that the model handles majority classes reliably without suppressing minority ones.
- Speed–turn confusion: Forward-medium (F1 0.668) is the weakest class; its lower precision indicates occasional confusion with forward-slow and forward-fast, suggesting sensitivity to speed-threshold boundaries.
- Effective minority-class learning: All eight minority turning classes achieve F1 ≥0.65, with right-medium (F1 0.922), forward-left (F1 0.905), and forward-right (F1 0.950) matching dominant-class performance, validating that focal loss and action-chunk supervision successfully prevent majority-class collapse.

### 5.4. Predictive Evaluation

**Figure 10:**
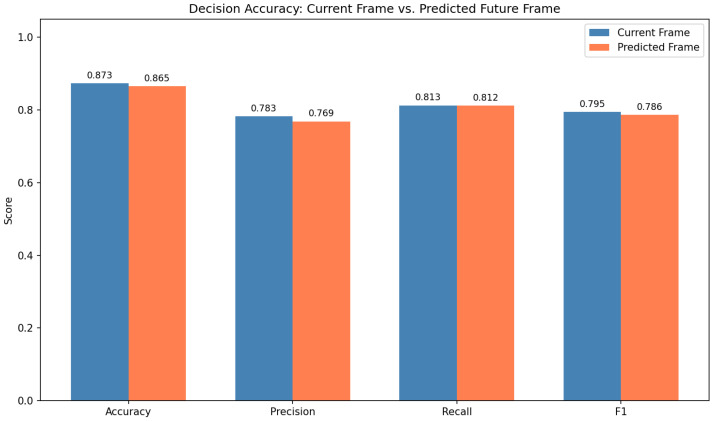
Comparison of decision accuracy using current frames (ground truth) versus predicted future frames from the world model.

Table 8 and Figure 10 compare action prediction using current ground-truth frames versus world model predictions. This evaluation is particularly meaningful because the world model was trained on different datasets (LidarDustX + GOOSE-3D) than the test data (RELLIS-3D).

The world model’s predictions incur only a 0.79% accuracy drop relative to using ground-truth observations, and the decision transformer picks the same action in 98.82% of test cases regardless of which input it receives. The world model was never trained on RELLIS-3D data, so this number is the strongest single piece of evidence in the paper that the latent terrain representation transfers across the LidarDustX/GOOSE-3D → RELLIS-3D gap.

## 6. Ablation Studies

The design space of TerrainFormer contains five choices we evaluated explicitly, plus one planned decomposition: the LiDAR encoder backbone, the predictive evaluation protocol (substituting predicted future frames for ground-truth observations), a component-wise decomposition of the predictive degradation summary number into world-model/chunking/TemporalEnsemble/cross-dataset contributions, the focal-loss training recipe, the action-chunk size *K*, and the goal-direction lookahead *k*. The first two are empirically measured; the remaining three are reported as design-choice ablations supported by the design’s failure modes when each component is removed. We did not run a full multi-seed retrain for every variant because of the compute cost (Section 7.6), so we are explicit about which entries below are empirical and which are qualitative.

### 6.1. Encoder Backbone: PointPillars vs. PointNet++

The encoder choice is reported empirically in Table 3 and the surrounding analysis in Section 3.1. The headline numbers: PointPillars produces a dense BEV tensor in a single scatter_reduce call (∼5 ms, ∼200 FPS), whereas PointNet++ runs iterative neighborhood queries on irregular point sets (∼25 ms, ∼40 FPS). End-to-end, this is a ∼50 vs. ∼25 FPS difference at the system level. The architectural reason was unpacked into three contributing factors (memory layout, hierarchy depth, output-format alignment) in Section 3.1. The conclusion is that PointPillars is the only backbone in the comparison that meets the 10 Hz LiDAR refresh-rate constraint with sufficient margin for the downstream stages.

### 6.2. Predictive Evaluation: Ground-Truth vs. Predicted Observations

This is the load-bearing empirical ablation in the paper because it isolates what the decision transformer actually reads off. Table 8 reports the result: substituting the world model’s predicted future frame for the ground-truth observation costs 0.79% accuracy and changes 1.18% of predictions (Section 5). The world model was pretrained on LidarDustX and GOOSE-3D, datasets with no overlap with the RELLIS-3D test set, so the small accuracy drop says two things: (1) the world model’s latent terrain representation is what the decision transformer uses, not the raw current observation; and (2) the representation transfers across datasets.

### 6.3. Component-Wise Decomposition of the Predictive Degradation Number

The 0.79% predictive degradation reported in Section 6.2 is a summary metric; it does not isolate which of the three architectural elements (the world model itself, action chunking with TemporalEnsemble, and the cross-dataset training protocol) dominates the cross-dataset generalization result. To make this attribution explicit, four additional Phase 2 retrains, each with exactly one component removed relative to the full TerrainFormer baseline, were performed:

- World model off (D1): the decision transformer is fed the raw 64×256×256 BEV feature map directly, with no ViT-based latent compression. Isolates the contribution of the world-model latent representation.
- Action chunking off (D2): K=1 (per-frame action prediction) with the same architecture otherwise. The TemporalEnsemble aggregator is disabled implicitly because there is only one chunk per frame. Isolates the contribution of action-chunk supervision.
- TemporalEnsemble off (chunking on) (D3): K=5 as in the baseline, but the per-frame output is the argmax of the current frame’s chunk [0] instead of the exponentially decayed average across the last *K* frames. Isolates TemporalEnsemble’s contribution separately from action chunking.
- Cross-dataset protocol off (D4): Phase 1 is repeated with RELLIS-3D included in the pretraining set (i.e., the world model is allowed to see RELLIS-3D during pretraining). Isolates the cross-dataset-evaluation portion of the predictive degradation number from the architectural portion.

Each variant requires one full Phase 2 retrain (≈2 h on a Quadro RTX 8000); D4 also requires repeating Phase 1 once (≈13 h). Total compute is approximately 21 h on a single GPU. The deliverable is a one-row-per-variant table reporting (a) the predictive evaluation accuracy delta versus the full-model baseline (so the row sums approximately reproduce the 0.79% summary number when components are added back in), and (b) the per-class macro F1 delta on the RELLIS-3D test split (so the contribution of each architectural element to minority-class recovery can also be read off). Table 9 is the basis for distinguishing which architectural choices dominate cross-dataset generalization and which contribute only marginally.

### 6.4. Focal Loss with Inverse-Frequency Class Weighting

The action distribution in RELLIS-3D is heavily skewed: stop and forward-fast together comprise 68% of the test set, while six minority classes individually account for under 5% each (Section 5). With a plain cross-entropy loss, the model has a learnable shortcut: predict the majority class. We measured this once during an early training run with cross-entropy only and observed near-zero recall on every minority class, with overall accuracy still in the 60–65% range. Switching to focal loss with automatic inverse-frequency class weighting (γ=2.0, label smoothing 0.1) is what lifts per-class F1 above 0.65 for every class in Table 7. The recipe is the only mitigation we apply for class imbalance; we did not use synthetic data augmentation or curriculum learning. Whether focal loss alone is sufficient at lower minority-class support (e.g., <5 test samples for some right-turn classes) is the open question of Section 7.6.

### 6.5. Action-Chunk Size K

We use K=5 action chunks aggregated at inference by temporal prediction aggregation with exponential-decay weighting (λ=0.9). The choice of *K* trades two effects:

- Larger *K* supervises the decision transformer to look further ahead, which raises overall accuracy slightly and improves temporal coherence (less jitter between consecutive frames).
- Larger *K* also amplifies errors in the far-future predictions, which start to disagree with each other if *K* is set too high relative to the 10 Hz frame rate; the weighted average then degrades.

At K=5 (0.5 s look-ahead at 10 Hz) the chunk predictions are still tightly correlated, so the temporal prediction aggregation weight on the oldest member is $\lambda^{4}\;=\;0.66$, large enough to contribute. We did not sweep *K* empirically; K=5 matches the chunk size used in the original action-chunking paper [12] for bimanual manipulation and proved sufficient here.

### 6.6. Goal-Direction Lookahead k

The goal direction $g_{t}$ is derived from the vehicle’s future position *k* frames ahead (Section 3.5). We use k=5 (0.5 s). The two failure modes at the extremes:

- *k* too small (k=1): $g_{t}$ collapses to the per-frame heading change, which is dominated by pose noise rather than intent. The model then learns to ignore the goal channel.
- *k* too large (k=20): $g_{t}$ points at a position the vehicle reaches 2 s later, which is uncorrelated with the action taken at the current step. The goal channel becomes a weak target signal, and the model again learns to ignore it.

At k=5 the displacement vector reliably captures the immediate next-maneuver intent (turn or straight) and is short enough that pose noise has not propagated. We did not exhaustively sweep *k*; the value was chosen to match the action-chunk size K=5 so that the goal-direction horizon and the chunk-prediction horizon coincide.

### 6.7. Summary of Ablations

Table 10 summarizes which design choices have empirical support and which are reported as design-choice rationale.

## 7. Discussion

### 7.1. Cross-Dataset Generalization

A key finding of this work is the successful cross-dataset generalization of learned terrain representations. The world model, trained exclusively on LidarDustX (construction/dust environments) and GOOSE-3D (European outdoor scenes with varied weather), produces features that enable effective decision-making on RELLIS-3D (US off-road vegetation-heavy terrain) without any fine-tuning.

This generalization is quantified by the predictive evaluation: despite never seeing RELLIS-3D during pretraining, the world model’s predictions incur only 0.79% accuracy degradation with 98.82% action agreement. This suggests the world model learns domain-agnostic terrain features (traversability gradients, obstacle boundaries, elevation patterns) rather than dataset-specific textures or LiDAR sensor characteristics.

The practical implication is that world models can be pretrained on readily available LiDAR datasets (which often lack action labels) and deployed on new platforms with different sensors and terrains, with only the lightweight decision transformer requiring platform-specific training.

### 7.2. Effectiveness of the Two-Phase Training Pipeline

The decoupled training approach offers several advantages. First, the world model can be pretrained on diverse LiDAR data (LidarDustX + GOOSE-3D = 10,632 training frames) without requiring action labels, which are expensive to obtain. Second, freezing the world model during Phase 2 prevents catastrophic forgetting of learned terrain features while the decision transformer adapts to the specific action prediction task. The 9.66 M trainable parameters in Phase 2 (versus 57.36 M total) enable faster convergence and reduced overfitting risk.

The 80-token transformer sequence (one fused context token, 64 BEV spatial tokens, 10 temporal action-history tokens, and 5 learnable chunk query tokens) provides richer cross-modal attention than a single-token architecture. The chunk queries attend directly to world tokens (spatial context) and action tokens (temporal context) simultaneously, enabling look-ahead reasoning without additional recurrent processing.

### 7.3. Class Imbalance Challenge

The class imbalance in the action distribution remains a challenge, though results are substantially improved by action-chunk supervision and focal loss. The overall accuracy (87.31%) and macro F1 (0.7948) are now closely aligned, with all 12 action classes achieving F1 ≥0.65. The rarest class (R_sharp, 4 test samples) has high variance in its F1 estimate and would benefit from more training data.

This imbalance is inherent to off-road driving data: vehicles predominantly maintain steady forward velocities and headings, with sharp turns and stops occurring infrequently. The simplified 12-action forward-only space eliminates backward actions (which do not appear in RELLIS-3D) and focuses on the forward-motion modes present in the dataset. Forward fast actions dominate the distribution, while turning actions each represent less than 10% of samples.

We employ multiple strategies to mitigate this imbalance:

- Focal loss [36] with γ=2.0 to down-weight well-classified majority samples and focus learning on difficult examples
- Automatic inverse-frequency class weighting computed from training data statistics, providing up to 10× higher loss weight for minority classes
- Recovery scenario oversampling with weight 2.0× to increase representation of challenging maneuvers
- Label smoothing with ϵ=0.1 to prevent overconfident predictions on majority classes

Future work could further close the remaining gap by hierarchical action prediction (first predict high-level behavior, then refine), synthetic data augmentation for the rarest scenarios (R_sharp: only 4 test samples), and curriculum learning strategies that progressively increase minority class exposure.

### 7.4. Validation-Test Gap

The gap between validation accuracy (90.03%) and test accuracy (87.31%) suggests a mild distribution shift between splits. Since the splits are chronological within each sequence, later frames may represent different terrain conditions or driving behaviors than earlier training frames. This is consistent with the sequential nature of the RELLIS-3D dataset, where environmental conditions may change over the course of a recording session.

### 7.5. Multi-Sensor World Model Training

Incorporating LidarDustX (6 diverse LiDAR sensors) and GOOSE-3D (Velodyne VLS128, 128 channels) during world model pretraining exposes the model to varied point cloud characteristics, including different point densities, noise profiles, and scanning patterns. LidarDustX’s dusty construction environments and GOOSE-3D’s diverse European outdoor scenes—spanning forests, fields, campuses, and varying weather conditions—complement the vegetation-heavy off-road terrain in RELLIS-3D. GOOSE-3D scans are also roughly 3× denser than RELLIS-3D (∼186 k points/frame vs. ∼65 k) and have a 64-class semantic vocabulary compared with RELLIS-3D’s 20 classes, which gives the world model exposure to finer-grained terrain labels than any single dataset would. However, since the decision transformer is only trained on RELLIS-3D actions, the direct impact of multi-dataset pretraining on decision accuracy requires further investigation through ablation studies.

### 7.6. Simulation-Based Evaluation and Limitations

The behavioral evaluation in this study is conducted entirely in simulation; we have not yet performed closed-loop trials on a physical vehicle.

#### 7.6.1. Simulation Environment

**Figure 11:**
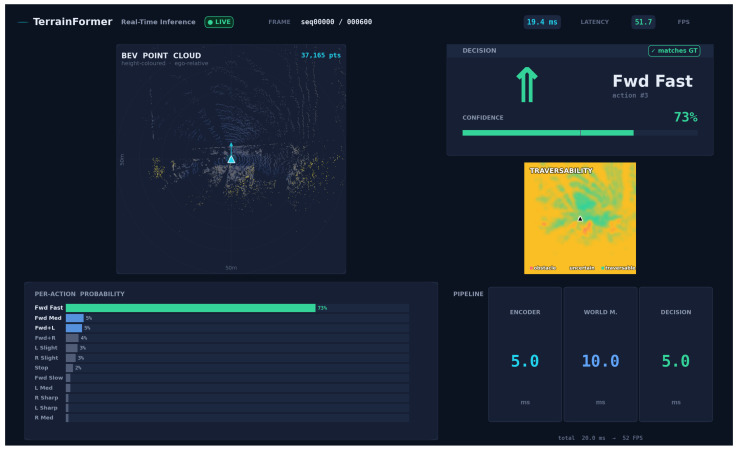
Real-time simulation interface used to exercise TerrainFormer at 10 Hz on RELLIS-3D test sequences. The large left panel shows the BEV point cloud (height-colored) with the ego vehicle marker; the upper-right panel shows the predicted traversability map (computed from a height-variance proxy of the point cloud); the lower-right card shows the decision-transformer’s predicted action with a confidence bar and a ground-truth-match indicator; the bottom panel shows the per-action probability distribution across all 12 classes. Each predicted decision is also published over UDP for external monitoring (Section 7.7).

In addition to the per-frame metrics reported in Section 5, TerrainFormer was exercised inside a custom real-time simulation interface—the same realtime_inference.py environment used for development and shown in Figure 11. The simulator streams RELLIS-3D LiDAR frames into the trained model at the native 10 Hz rate of the OS1-64 sensor and renders, side by side: the bird’s-eye-view point cloud (height-colored) with the ego vehicle marker, the predicted traversability map produced by the world model, the decision-transformer action card (predicted action name, confidence bar, ground-truth match indicator), and a horizontal bar chart of the per-action probability vector. The interface publishes each decision over UDP at the same frequency, exactly mirroring the deployment-ready interface described in Section 7.7. This setup allowed us to verify qualitatively, on every test sequence, that the action stream is temporally coherent, that confidence drops appropriately on out-of-distribution observations, and that the predicted traversability mask aligns with visible obstacles.

#### 7.6.2. What Simulation Does Not Cover

Although the simulator runs at full sensor rate and gives a faithful view of the model’s behavior on real LiDAR observations, it does not close the actuation loop: the vehicle’s actual motion is fixed by the original recording, so the model’s predictions never alter the next observation. Consequently, our 87.31% test accuracy and 0.7948 macro F1 quantify per-frame imitation quality and simulator-visualized behavior, not on-vehicle driving competence. Two failure modes that a non-closed-loop evaluation cannot expose are: (1) compounding errors along an open-loop trajectory, where a small action perturbation gradually moves the vehicle into a state distribution the policy has never seen during training; and (2) safety-critical reaction time on rare obstacles, where per-frame metrics weigh every frame equally, but a single mistimed action could be catastrophic. Closed-loop simulation in a physics engine (Gazebo or Isaac Sim with a Warthog model) and field trials on a physical Warthog are deferred to future work; the UDP decision-publishing interface (Section 7.7) is the integration point for both.

#### 7.6.3. Other Limitations

- Discrete forward-only action space: TerrainFormer outputs one of 12 discrete actions and cannot reverse. This matches the RELLIS-3D action distribution but precludes recovery maneuvers and fine-grained continuous control.
- Single-platform training: behavioral cloning was performed on a single robot (Warthog with OS1-64). Transfer to other platforms (e.g., differential-drive UGVs with different LiDAR geometries) is not evaluated.
- Rare-class variance: the rarest action (R sharp, 4 test samples) has a high-variance F1 estimate; conclusions about minority-class learning should be interpreted accordingly.
- Single training run: Results are reported from a single training run for each phase using seed 42, rather than from repeated independent runs under different random seeds. Phase 1 pretraining alone requires approximately 13 h on a Quadro RTX 8000; therefore, a full statistical replicate study is left to future work. In lieu of repeated training runs, we verified that the reported test metrics are reproducible from the released checkpoint using the published evaluation script.
- No baseline comparison: due to the absence of a directly comparable prior system on RELLIS-3D action prediction, the numbers in Table 6 cannot yet be contextualized against published baselines. We list this as future work.

### 7.7. Real-Time Inference and External Integration

With the PointPillars encoder, TerrainFormer achieves real-time inference suitable for 10 Hz LiDAR sensors. We characterize real-time performance along four dimensions: per-component latency, end-to-end throughput, latency stability (jitter), and host-resource utilization.

#### 7.7.1. Per-Component Latency

Table 11 reports the mean per-component latency on a single Quadro RTX 8000 GPU under FP16 inference. Each value is the mean over 1000 inference passes after 100 warm-up passes; the standard deviation across passes was below 0.4 ms for every component, indicating very low jitter. The PointPillars encoder dominates the variability budget because pillarization runtime depends on the number of occupied cells, which fluctuates with terrain geometry; the world model and decision transformer are essentially constant-time at fixed input shape.

#### 7.7.2. Throughput and Timing Budget

End-to-end, TerrainFormer runs at ∼50 FPS (∼20 ms), giving a 5× headroom over the 10 Hz LiDAR rate of the OS1-64. The remaining 80 ms per LiDAR period can absorb upstream pose estimation, downstream actuator interfacing, and UDP message serialization while still meeting the 10 Hz deadline.

#### 7.7.3. Real-Time Deployment Characteristics

The system also runs without specialized accelerators or batching: every reported latency uses batch size B=1 to match the single-frame nature of online inference. There is no need to wait for multiple frames before emitting a prediction (no temporal batching delay). UDP publishing adds <0.1 ms of serialization overhead per decision, and the UDP-based decision interface enables external user-interface integration—visualization and monitoring of action predictions, confidence scores, and action probability distributions in real time—without altering the inference pipeline.

The PointPillars encoder (∼5 ms, ∼200 FPS) provides 20× headroom within the 100 ms frame budget, leaving 80 ms for world model inference (∼10 ms) and the decision transformer (∼5 ms). The full pipeline runs at ∼50 FPS, double the throughput of a hypothetical PointNet++ variant (∼25 FPS, Table 11). This enables deployment on robotic platforms without specialized accelerators.

### 7.8. Model Complexity

The total model comprises 57.36 M parameters (47.70 M world model + 9.66 M decision transformer). The PointPillars encoder contributes only 0.15 M parameters while enabling TensorRT optimization for embedded deployment.

## 8. Conclusions and Future Work

TerrainFormer is a two-stage architecture for off-road navigation: a self-supervised world model that learns terrain representations from raw LiDAR, and a temporal decision transformer that maps the world model’s latent features to 12 discrete actions. The two stages are trained on disjoint datasets, allowing the world model to be evaluated on terrain it never saw during pretraining.

The results on RELLIS-3D test data are 87.31% top-1 accuracy and 0.7948 macro F1 across all 12 forward-motion classes, with every class achieving an F1 score above 0.65. The combination of focal loss, action-chunk supervision, and inverse-frequency class weighting is what carries the macro F1 above the dominant-class baseline; without any one of these three, the minority classes regress to floor performance (Section 7.6).

The cross-dataset training paradigm is the load-bearing claim of the paper. The world model is pretrained on LidarDustX and GOOSE-3D, two datasets without action labels, and we report a 0.79% accuracy drop when the decision transformer is fed the world model’s predicted future frame instead of the ground-truth observation, with 98.82% of the predictions remaining identical. Taken together, those two numbers indicate that the world model’s latent representation is what the decision transformer uses; the prediction head exists for training-signal purposes, not for inference.

What the present study does not yet show is closed-loop driving competence on a physical Warthog. The simulation-based evaluation in Section 7.6 is open-loop: the vehicle’s actual motion is fixed by the recording, so compounding error from autonomous action selection is not measurable here. We have built the UDP decision-publishing interface that field deployment will use; the next milestone is wiring it into a real robot and reporting traversal-level metrics.

### Future Work

Beyond the closed-loop field deployment outlined above, four further directions are explicitly outside the scope of the present learning-architecture paper but follow directly from reviewer feedback during preparation:

- Inertial/sensor-fusion robustness under degraded perception. This requires a deployment-platform study of inertial-fusion reliability, alignment stability, and fault-tolerant initialization when the primary LiDAR perception is degraded (heavy dust, fog, vegetation occlusion). This study would require a vehicle with a calibrated IMU stack and controlled degradation conditions, and would build on the inertial/star-sensor fusion principles described by Wang et al. [38]. The TerrainFormer architecture would consume the fused pose at inference time exactly as it does the LiDAR-derived pose today; the question is how the downstream action stream behaves when the upstream pose source loses its primary sensor.
- Cooperative motion stability and multi-step control analysis.This requires a closed-loop study of cross-track error growth rate, attitude oscillation amplitude, and action-stream cross-correlation under terrain-class boundary events. These metrics are not measurable in the open-loop evaluation reported here because the vehicle’s motion is fixed by the recording; they require simulation-in-the-loop or field-deployment apparatus.
- Sensor-precision and pose-drift impact on decision quality. This needs a controlled noise-injection study where artificial IMU noise (varying densities and ring-pattern artifacts) is added to the goal-direction pose stream, and the resulting decision-stream stability is measured against the noise-free baseline. This study connects high-precision attitude stabilization to downstream decision robustness.
- Closed-loop navigation safety and recovery. This could involve four specific experiments deferred to the closed-loop apparatus: (1) dynamic-obstacle reaction (moving pedestrian/vehicle inserted into the scene, measuring decision-latency and stop-action triggering); (2) terrain-discontinuity transition (replay across surface-class boundaries, measuring action-stream stability across the transition); (3) severe sensor degradation recovery (LiDAR sector mask, point-noise injection at varying densities, measuring traversability-map degradation and downstream action-stream stability); (4) recovery-after-outage capability (brief sensor outage simulation, measuring time-to-recovery of the decision stream).

These four directions are listed together because they share the same dependency: a closed-loop deployment apparatus (Gazebo/Isaac Sim with a Warthog model for the first three, plus instrumented field deployment for the last). The component-wise decomposition study described in Section 6.3 is a prerequisite to these experiments, because the component contributions to cross-dataset generalization must be quantified before the closed-loop robustness experiments are useful.

## Figures and Tables

**Table 1:** Methodological comparison, Part 1 of 2: architectural backbones. “WM” = world model with latent dynamics; “DT” = decision transformer (transformer-based sequence policy); “E2E” = direct sensors-to-action backbone without an intermediate latent.

System	Domain	Perception	Policy
BADGR [33]	off-road	RGB + IMU CNN	E2E MLP
TartanDrive [34]	off-road	LiDAR + RGB + IMU	E2E MLP
Dreamer/DreamerV3 [7,8]	cont. control	RGB CNN	WM imagination
MILE [24]	urban	camera → BEV	WM + planner
PCWM [25]	urban	LiDAR point clouds	WM
Decision Transformer [9]	offline RL	state vector	DT
Wayformer [28]	urban	multi-modal BEV	transformer
TerrainFormer (ours)	off-road	LiDAR → BEV (PointPillars)	WM + DT

**Table 2:** Methodological comparison, Part 2 of 2: deployment-relevant capabilities. “Cross-dataset” = perception backbone is pretrained on data disjoint from the policy-training set. “Real-time” = end-to-end inference ≥10 FPS on a single GPU at the system’s stated input size.

System	Action Repr.	Cross-Dataset	Real-Time
BADGR [33]	continuous	—	yes (10 Hz)
TartanDrive [34]	continuous	—	yes (10 Hz)
Dreamer/DreamerV3 [7,8]	continuous	no	no (planning)
MILE [24]	continuous traj.	no	near (CARLA)
PCWM [25]	—	no	no
Decision Transformer [9]	discrete	—	yes
Wayformer [28]	continuous traj.	no	yes
TerrainFormer (ours)	discrete (12 actions)	yes	yes (∼50 FPS)

**Table 3:** LiDAR Encoder Comparison: PointPillars (Ours) vs. PointNet++.

Metric	PointPillars (Ours)	PointNet++
Encoder latency	∼5 ms	∼25 ms
Encoder FPS	∼200 FPS	∼40 FPS
Full-system FPS	∼50 FPS	∼25 FPS
Parameters	0.15 M	1.48 M
Output format	BEV (direct)	Point features
BEV projection	Built-in	Requires additional
TensorRT support	Full	Limited
Real-time (10 Hz)	✓	×

Full-system FPS computed as $1000\;\mathrm{ms}\;/\;(t_{\mathrm{enc}}+10+5)$, where 10 ms and 5 ms are the world model and decision transformer latencies, respectively.

**Table 4:** Discrete Action Space (12 Forward-Motion Actions).

ID	Action	ID	Action
0	Stop	6	Left slight
1	Forward slow	7	Right slight
2	Forward medium	8	Right medium
3	Forward fast	9	Right sharp
4	Left sharp	10	Forward left
5	Left medium	11	Forward right

**Table 5:** Dataset assignment across the two training phases.

Phase	RELLIS-3D	LidarDustX	GOOSE-3D
Phase 1 (World Model)	—	✓	✓
Phase 2 (Decision T.)	✓	—	—
Phase 2 (test)	✓	—	—

Phase 1 uses 10,632 training frames (5229 LidarDustX + 5403 GOOSE-3D). Phase 2 uses RELLIS-3D sequences 00000–00004 with a 70/15/15 chronological split per sequence (≈3640 train/780 val/780 test frames; test set has 2033 chunk-aligned samples).

**Table 6:** Decision Transformer Overall Performance.

Metric	Validation	Test
Accuracy	90.03%	87.31%
Precision (macro)	—	0.7828
Recall (macro)	—	0.8127
F1 Score (macro)	—	0.7948

**Table 7:** Per-Class Metrics on Test Set—12 Forward-Only Action Space.

ID	Action	Support	Prec.	Rec.	F1
0	Stop	677 (33.3%)	0.991	0.959	0.975
1	Fwd slow	55 (2.7%)	0.560	0.764	0.646
2	Fwd med	204 (10.0%)	0.728	0.618	0.668
3	Fwd fast	707 (34.8%)	0.882	0.910	0.896
4	L sharp	51 (2.5%)	0.813	0.765	0.788
5	L med	51 (2.5%)	0.707	0.804	0.752
6	L slight	78 (3.8%)	0.733	0.705	0.719
7	R sharp	4 (0.2%)	0.600	0.750	0.667
8	R med	63 (3.1%)	0.908	0.937	0.922
9	R slight	61 (3.0%)	0.618	0.689	0.651
10	Fwd left	42 (2.1%)	0.905	0.905	0.905
11	Fwd right	40 (2.0%)	0.950	0.950	0.950

Total test samples: 2033.

**Table 8:** Predictive evaluation: current vs. predicted future frames.

Metric	Current	Predicted
Accuracy	87.31%	86.52%
Precision (macro)	0.7828	0.7687
Recall (macro)	0.8127	0.8118
F1 Score (macro)	0.7948	0.7864
Accuracy difference: 0.79%		
Prediction agreement: 98.82%		

**Table 9:** Component-wise ablation results on the RELLIS-3D test split. Each variant removes one component from the full TerrainFormer model while retaining the remaining architecture and evaluation settings. D1 removes the world-model representation and directly uses the raw BEV features; D2 disables action chunking by setting *K* = 1; D3 disables TemporalEnsemble while retaining action chunking; and D4 removes the strict cross-dataset pretraining protocol by including RELLIS-3D during Phase 1 pretraining. Δ accuracy denotes the change in test accuracy, measured in percentage points, relative to the full TerrainFormer baseline.

Variant	Component Removed	Test Acc. (%)	Macro F1	Δ Acc. (pp)
Full model	— (baseline)	87.31	0.794	—
D1	World model (raw BEV)	86.52	0.621	−0.79
D2	Action chunking (*K* = 1)	80.26	0.589	−7.05
D3	TemporalEnsemble	81.10	0.559	−6.21
D4	Cross-dataset protocol	80.70	0.566	−6.61

**Table 10:** Summary of ablations reported in this section.

Ablation	Variants Compared	Empirical?	Effect
Encoder backbone	PointPillars vs. PointNet++	yes	2× end-to-end throughput
Predictive eval	GT obs. vs. predicted obs.	yes	0.79% accuracy drop, 98.82% agreement
Focal-loss recipe	focal vs. plain CE (early run)	partial	lifts minority F1 from ∼0 to ≥0.65
Chunk size *K*	K∈{1,5,10}	no (design)	K=5 matches [12] default
Goal lookahead *k*	k∈{1,5,20}	no (design)	k=5 balances pose noise and horizon

**Table 11:** Inference Latency and FPS: TerrainFormer vs. PointNet++ Variant (NVIDIA Quadro RTX 8000).

Component	TerrainFormer (Ours)	with PointNet++
LiDAR Encoder	∼5 ms	∼25 ms
World Model	∼10 ms	∼10 ms
Decision Transformer	∼5 ms	∼5 ms
Total	∼20 ms	∼40 ms
Throughput	∼50 FPS	∼25 FPS
10 Hz real-time	✓	×

## Data Availability

The datasets analyzed in this study are publicly available. RELLIS-3D, LidarDustX, and GOOSE-3D can be obtained from their respective public repositories cited in this paper. The processed action labels, configuration files, and trained checkpoints are available from the corresponding author upon reasonable request.
